# Correction to ‘Decoding and encoding models reveal the role of mental simulation in the brain representation of meaning’

**DOI:** 10.1098/rsos.201162

**Published:** 2020-08-12

**Authors:** David Soto, Usman Ayub Sheikh, Ning Mei, Roberto Santana

*R. Soc. Open Sci.* **7**, 192043. (Published Online 20 May 2020) (doi:10.1098/rsos.192043)

(1) ‘Interval of 3.4 s and 6.8 s’ was wrongly used in place of ‘interval of 3.4 s and 8.6 s’. This has now been corrected.

(2) There is an error in the visualization of the informational connectivity results (Figure 5). The order of the ROI labels in the panel of the Figure were incorrect. Both Figure 5 and the Python script used to produce it have been updated. The Results in the main text have also been updated.

“There were two substrates that showed the strongest changes in informational connectivity with the rest of the semantic network. First, the fusiform gyrus showed increased informational connectivity with parahippocampal gyrus, middle temporal lobe, inferior parietal lobe, posterior cingulate gyrus, precuneus, lateral orbitofrontal cortex, pars opercularis, pars orbitalis, pars triangularis and superior frontal gyrus. A similar effect involved the left inferior temporal lobe, which showed increased connectivity during mental stimulation with fusiform gyrus, middle temporal lobe, inferior parietal lobe, precuneus, pars opercularis, pars triangularis and superior frontal gyrus. Figure 5 also shows that anterior temporal lobe and frontal pole showed no changes in informational connectivity as function of the depth of processing.”

(3) The finding previously reported that mental simulation changes the level of informational connectivity within the semantic network remains therefore unaltered. However, in the Discussion it is noted that

“Remarkably, the fusiform gyrus showed increased informational connectivity with multiple association areas, including default-mode regions and frontoparietal cortex, suggesting that the interplay between domain-specific and domain-general regions at a representational level, underlies mental simulation.”

**Figure 5.**
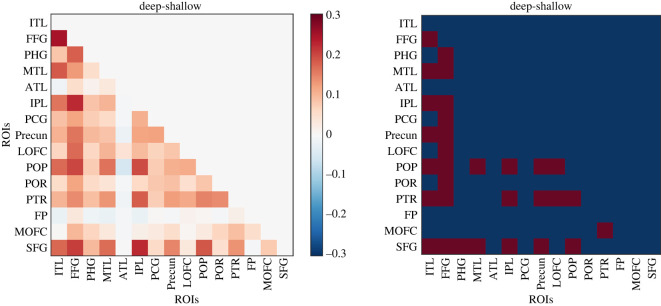
The figure shows the mean difference of informational connectivity between deep and shallow conditions. The right panel shows which of the pairs of ROIs were significantly more connected in deep as compared with the shallow condition (FDR corrected for multiple comparisons).

