# Supplementary material for: Decoding and encoding models reveal the role of mental simulation in the brain representation of meaning
Source: R Soc Open Sci. 2020 May 20;7(5):192043. doi: 10.1098/rsos.192043 (PMC7277265; doi:10.1098/rsos.192043)
Supplement: Supplemental Results [file rsos192043supp1.pdf]

Supplementary Materials

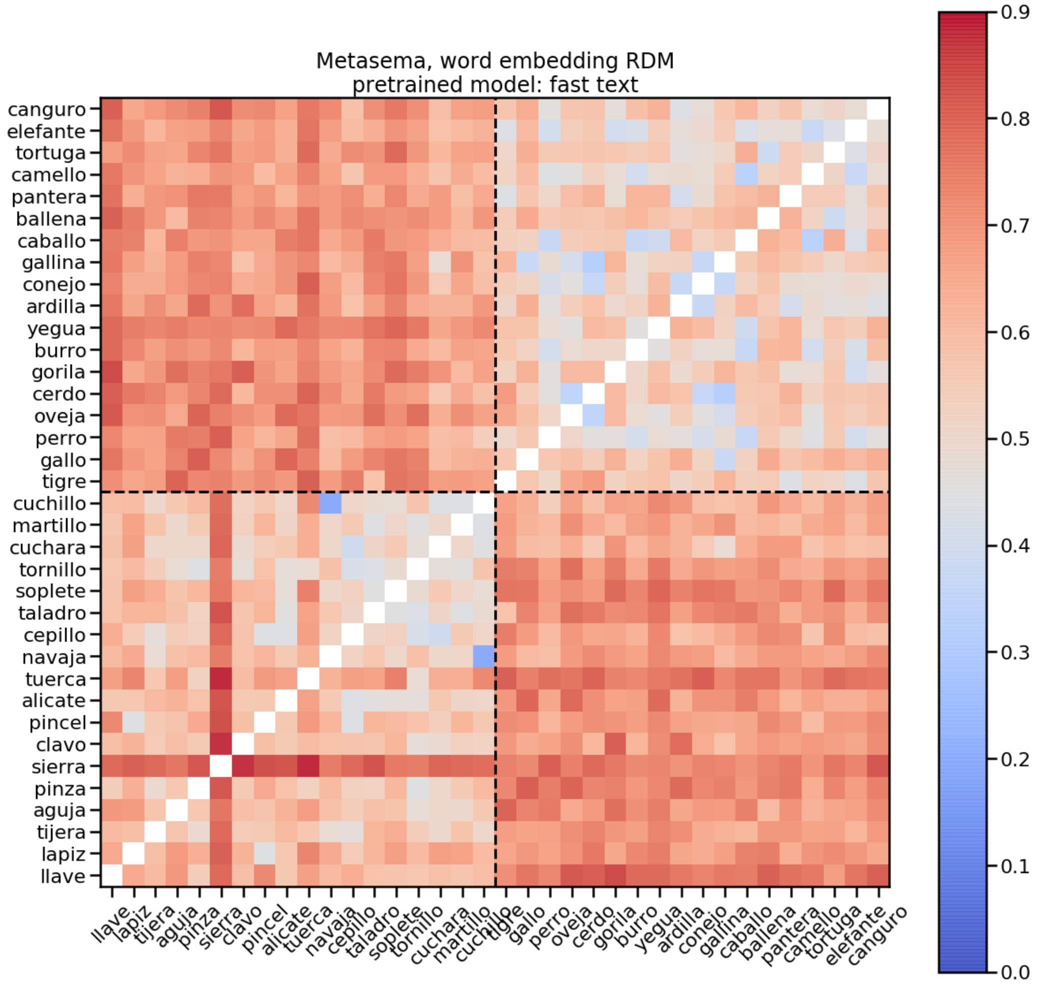

Figure S1: Representational Dissimilarity Matrix of Fast Text embedding features with cosine distance as metric.

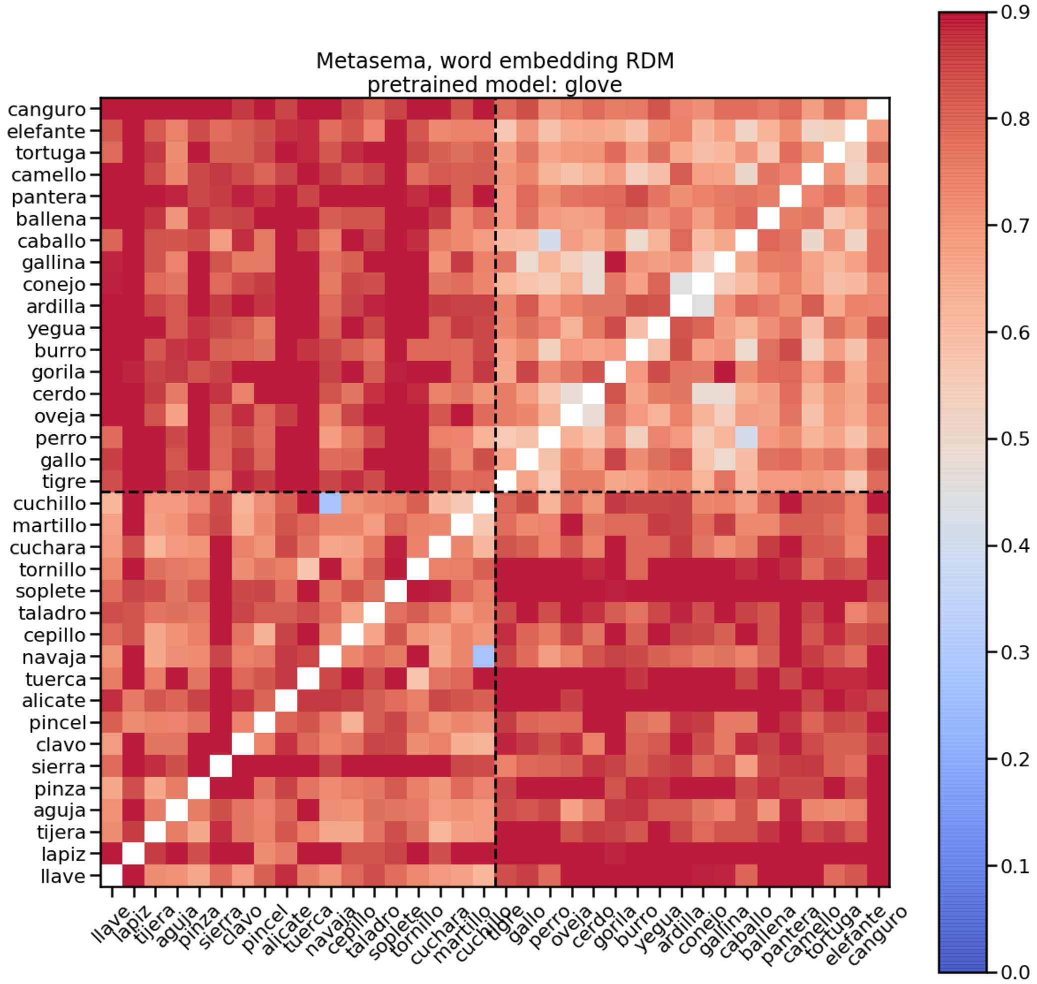

Figure S2: Representational Dissimilarity Matrix of GloVe embedding features with cosine distance as metric.

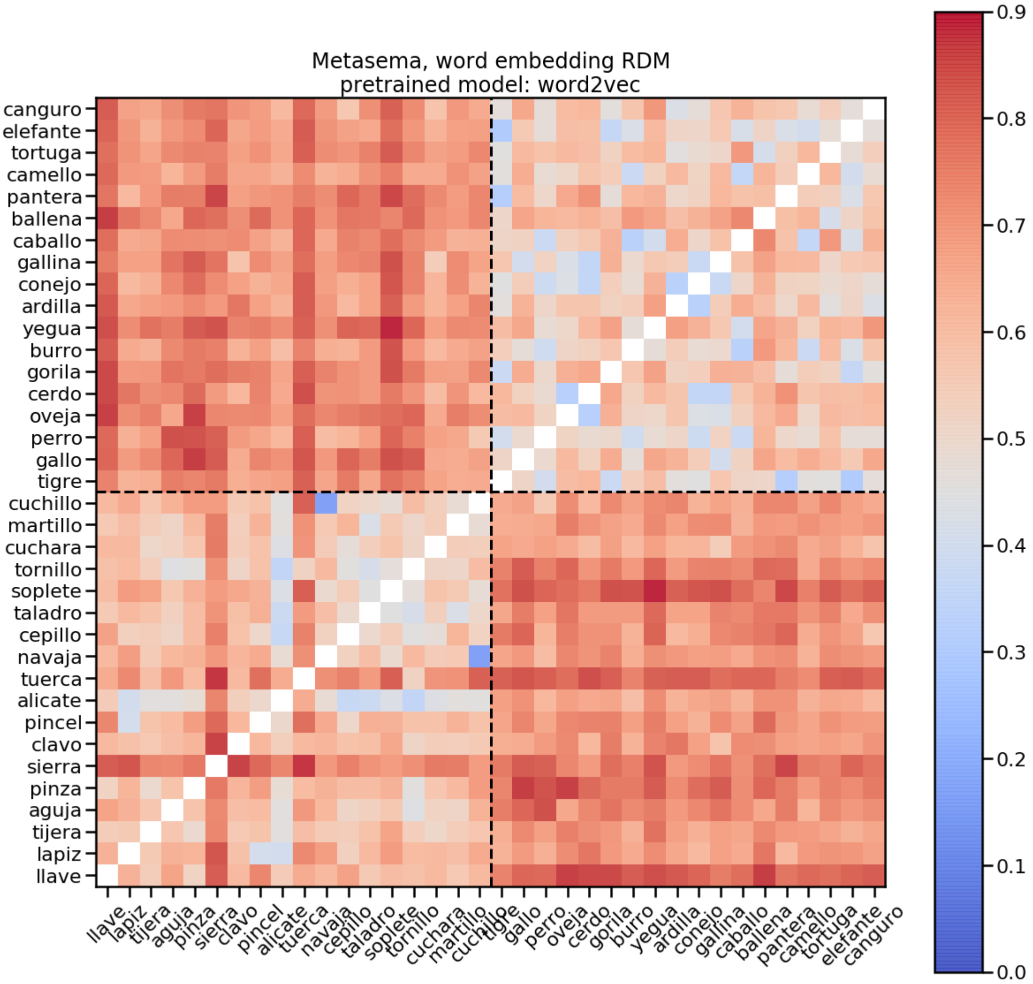

Figure S3: Representational Dissimilarity Matrix of Word2Vec embedding features with cosine distance as metric

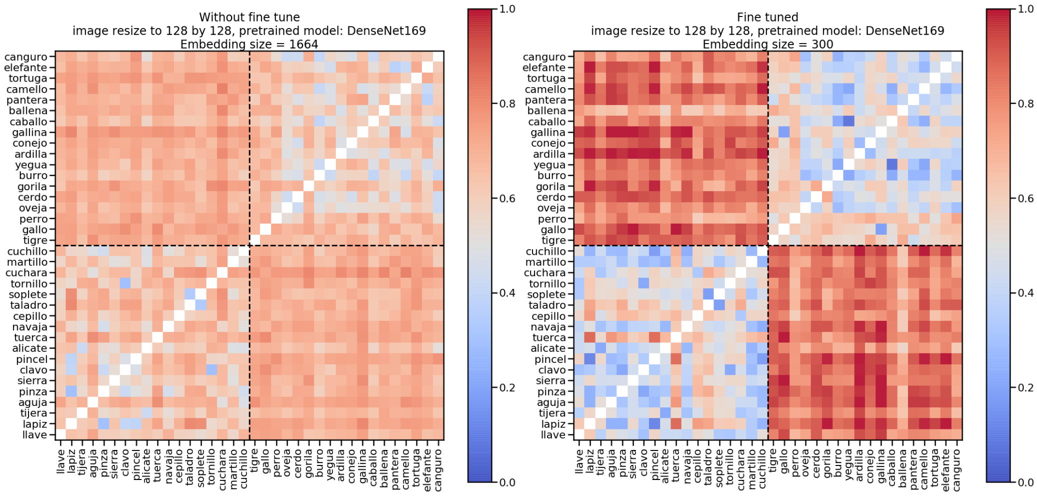

Figure S4: Representational Dissimilarity Matrix of DenseNet169 embedding features with cosine distance as metric.

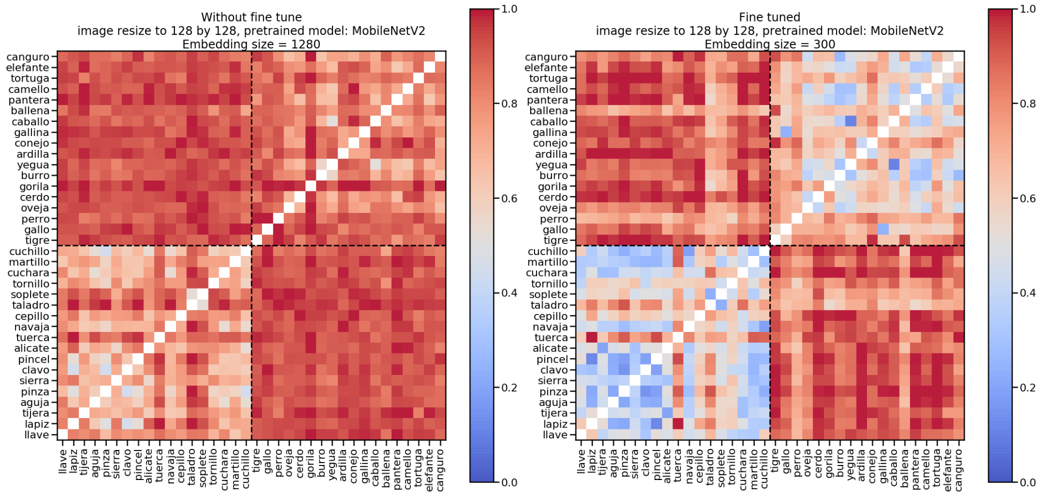

Figure S5: Representational Dissimilarity Matrix of MobileNetV2 embedding features with cosine distance as metric.

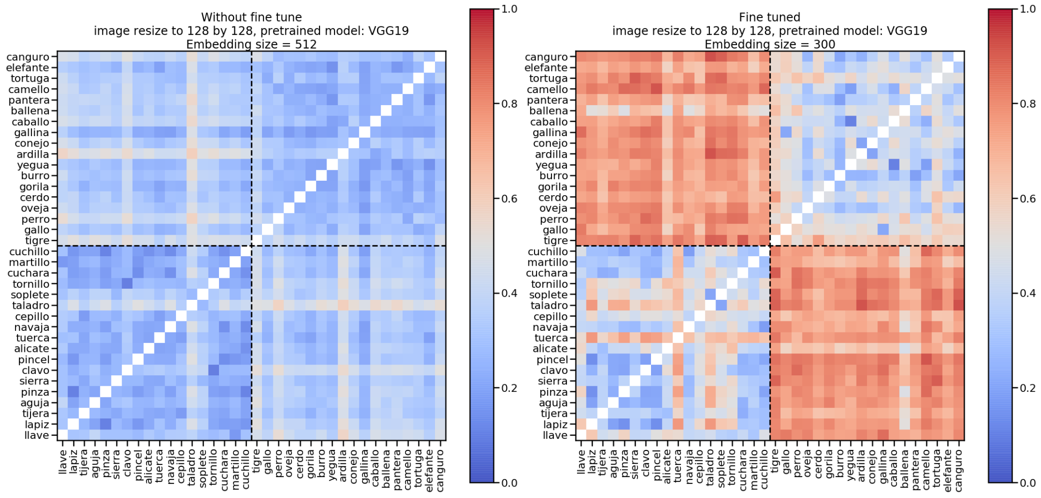

Figure S6: Representational Dissimilarity Matrix of VGG19 embedding features with cosine distance as metric.

Figure S7 illustrates the results of a leave-one-run-out cross-validation procedure. We found that classification of the semantic category was at chance-level in all pre-specified ROIs in the shallow processing condition, including the FP ($t(26) = 0.61$; $p = 0.78$), FFG ($t(26) = 0.92$; $p = 0.61$), IPL ($t(26) = 0.37$; $p = 0.82$), ITL ($t(26) = 1.78$; $p = 0.32$), LOFC ($t(26) = 0.28$; $p = 0.83$), MOFC ($t(26) = 1.30$; $p = 0.58$), MTL ($t(26) = 1.22$; $p = 0.58$), POP ($t(26) = 2.22$; $p = 0.26$), POR ($t(26) = 1.03$; $p = 0.59$), PTR ($t(26) = 0.22$; $p = 0.83$), PHG ($t(26) = -0.57$; $p = 0.78$), PCG ($t(26) = -0.44$; $p = 0.82$), Precun ($t(26) = 2.36$; $p = 0.26$), SFG ($t(26) = 1.95$; $p = 0.31$), ATL ($t(26) = -1.12$; $p = 0.59$). On the other hand, in the deep processing condition, the classification of the semantic category was found to be significantly above chance in all pre-specified ROIs: FP ($t(26) = 2.36$; $p = 0.026$), FFG ($t(26) = 8.16$; $p = 1.83e\text{-}07$), IPL ($t(26) = 7.40$; $p = 2.76e\text{-}07$), ITL ($t(26) = 6.78$; $p = 8.46e\text{-}07$), LOFC ($t(26) = 6.64$; $p = 1.04e\text{-}06$), MOFC ($t(26) = 3.63$; $p = 0.001$), MTL ($t(26) = 7.52$; $p = 2.75e\text{-}07$), POP ($t(26) = 6.23$; $p = 2.55e\text{-}06$), POR ($t(26) = 4.84$; $p = 7.04e\text{-}05$), PTR ($t(26) = 6.78$; $p = 8.46e\text{-}07$), PHG ($t(26) = 7.60$; $p = 2.75e\text{-}07$), PCG ($t(26) = 4.19$; $p = 0.0004$), Precun ($t(26) = 6.16$; $p = 2.72e\text{-}06$), SFG ($t(26) = 5.44$; $p = 1.58e\text{-}05$), and ATL ($t(26) = 2.60$; $p = 0.02$).

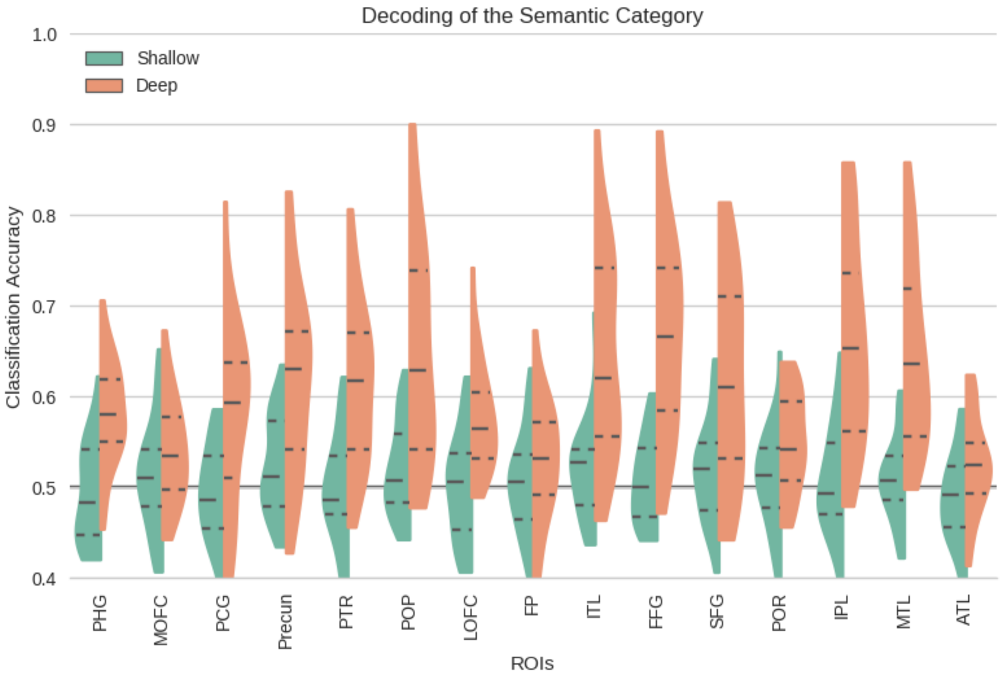

Figure S7: MVPA classification results obtained using leave-one-run-out cross-validation. Specifically, the Figure shows summary statistics of decoding accuracy for all pre-specified ROIs. The three dotted lines inside each violin are the quartiles. It can be seen that in the shallow processing condition, the decoding of the semantic category (living/non-living) was found to be at chance-level in all ROIs while in the deep condition, it was found to be above-chance and significantly better than the shallow condition (FDR corrected for multiple comparisons) in all ROIs.

Figure S8 shows the functional connectivity results, namely, the absence of differences in the temporal correlation between ROIs across the different conditions of processing depth.

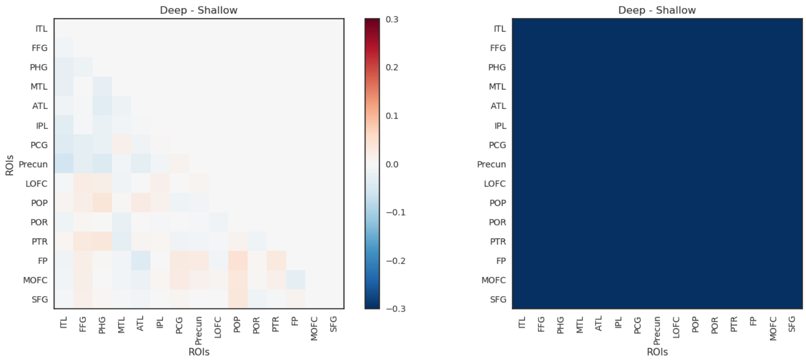

Figure S8: The left panel shows the mean difference of functional connectivity between deep and shallow conditions and the right panel shows that there were no pairs of ROIs for which this connectivity was found to be significantly different across the deep and shallow conditions. The functional connectivity between each pair of ROIs was calculated as follows. First, the fMRI data was preprocessed and prepared as presented in sections 2.4 and 2.6.1. Next, a time series was obtained for each ROI by taking the mean of all its voxels across each of the scans. To calculate the functional connectivity between the ROIs, these time-series were correlated using Pearson correlation for each of the pairs of ROIs, and a matrix of functional connectivity was created. This procedure was performed separately for shallow and deep processing sessions resulting in two matrices for each of the participants. Finally, to compare between the functional connectivity in deep and shallow conditions, a paired t-test was conducted with FDR correction.

Figure S9 shows the average variance explained by the computer vision (VGG19, Densent169, mobilenetV2) **without fine-tuning** and the word embedding (Fast Text, Glove, word2vec) models, averaged across 26 subjects. The errorbar represents 95% confidence interval of a bootstrapping with 1000 iterations. For each ROI, a one-way analysis of variance (ANOVA) was performed within the computer vision models and within the embedding models across subjects. The ANOVAs aimed to detect the difference in variance explained within a type of models. After all the ANOVAs were performed, FDR correction procedures were applied to the raw p-values to correct for the multiple comparison within each condition (deep v.s. shallow). There was no difference among different word embedding models, but there was a significant difference among the computer vision models in most of the ROIs for each condition. MobileNetV2, which was considered the simplest model within the computer vision models due to its smaller set of parameters, performed the best in explaining the variance in most of the ROIs for each condition.

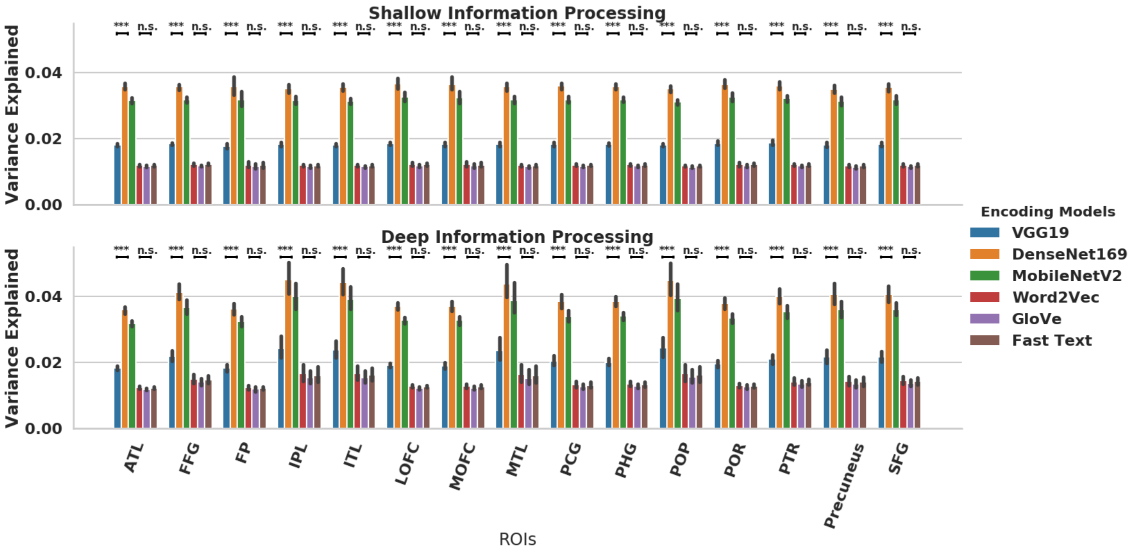

Figure S9: Average Variance Explained by each of the Word Embedding and Computer Vision Models. No fine-tuning was performed for the computer vision models.

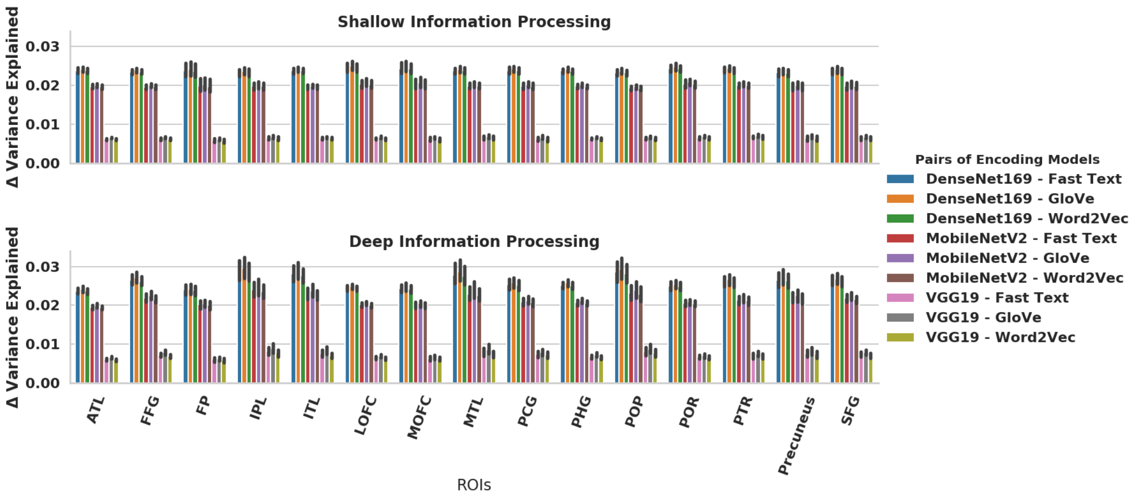

Figure S10: Differences between a Word Embedding and a Computer Vision model in variance explained. No fine-tuning was performed for the computer vision models.

We then computed the difference between each of the computer vision and word embedding models within each ROI and condition, in order to assess whether word embedding or computer vision models were better. One-sample t-tests against zero for each pair were conducted with FDR correction. All the computer vision models performed better than any of the word embedding models (see Figure S10).

Then, we computed the average difference between the computer vision models and the word embedding models. We then performed paired t-tests to compare the difference between the variance explained in the deep and shallow processing conditions, using FDR correction. Figure S11 illustrates the pattern of results within each ROI. We found that the advantage of computer vision models over word embedding was higher in the deep processing condition relative to the shallow processing in all ROIs in which there were statistically reliable differences.

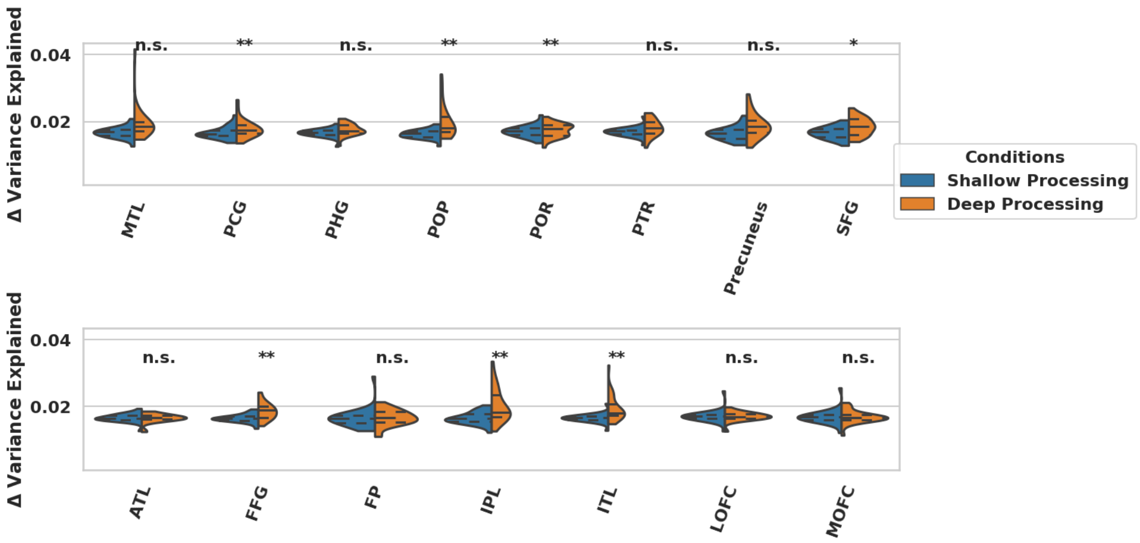

Figure S11: Overall Difference between Word Embedding and Computer Vision models per ROI. No fine-tuning was performed for the computer vision models.

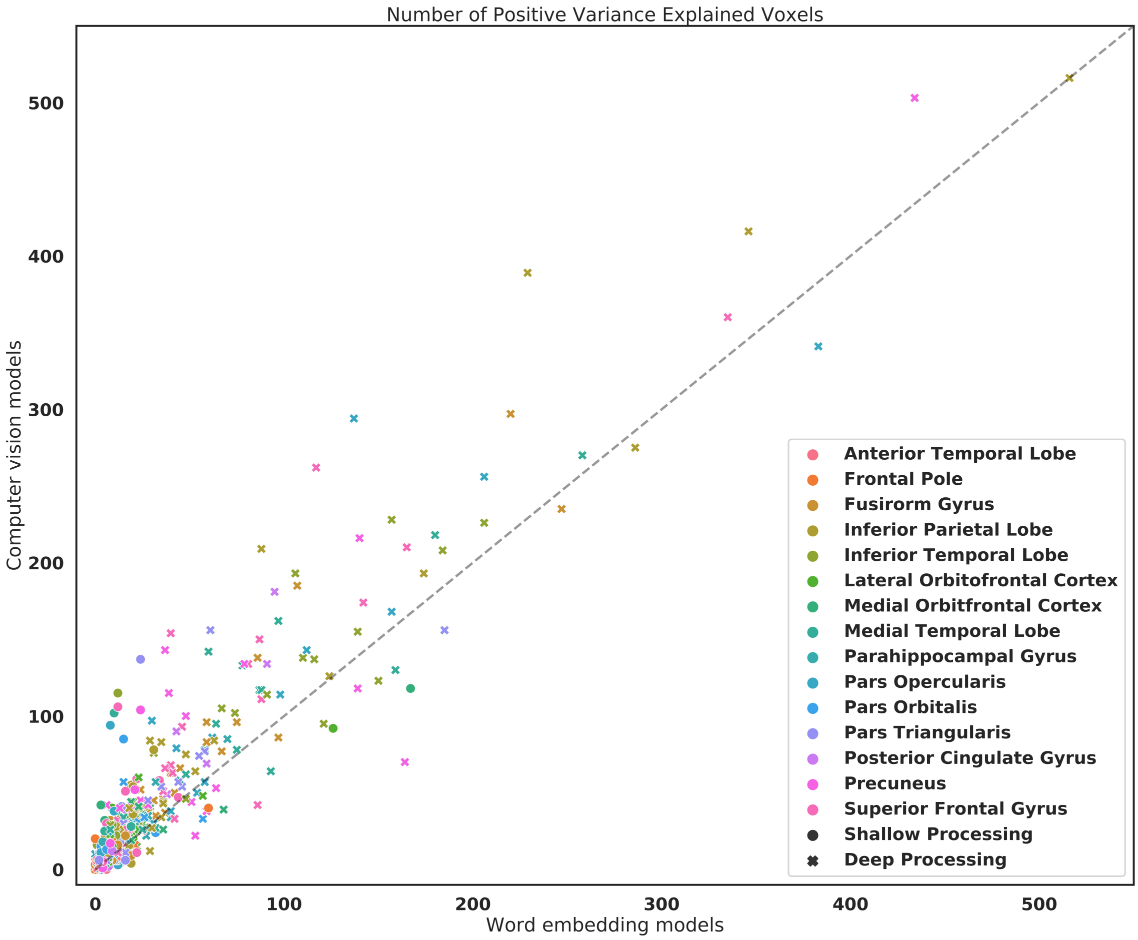

Figure S12: The comparison of number of positive variance explained voxels between the averages of word embedding and computer vision models. **x**:deep processing, **o**: shallow processing

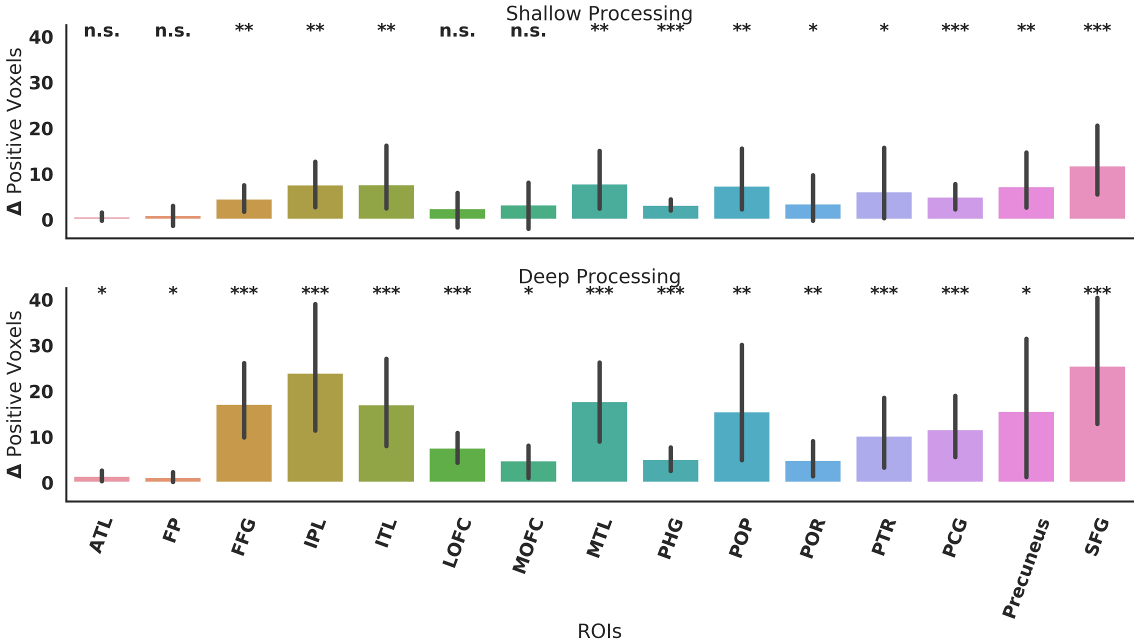

Figure S13: Difference of number of positive variance explained voxels between the averages of word embedding and computer vision models. One sample permutation t-tests were applied to estimate if the computer vision models explained more positive voxels than the word embedding models for a given ROI for a given condition (FDR corrected). Error bar represents bootstrapped standard error between subjects. n.s.: not significant, *: $p < 0.05$, **: $p < 0.01$, ***: $p < 0.001$

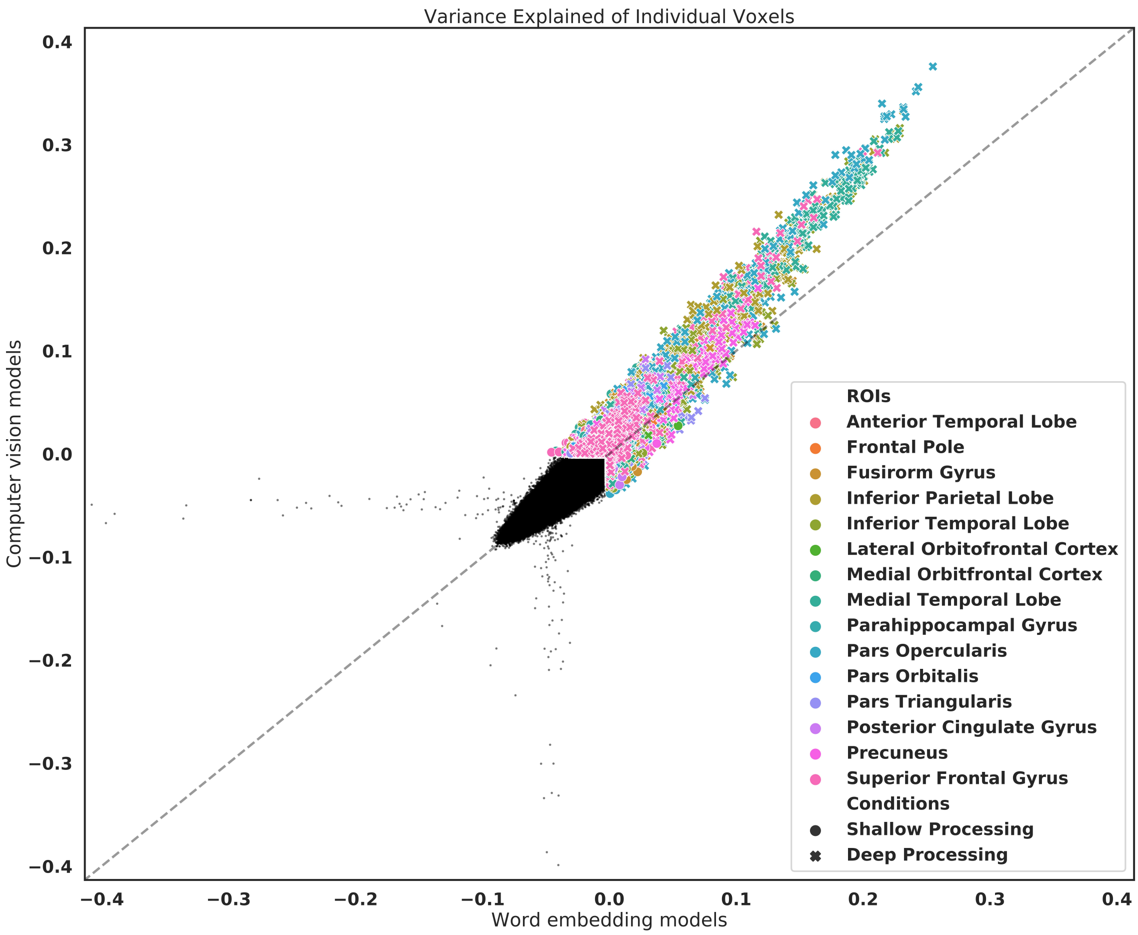

Figure S14: Comparison of variance explained of each voxel between the averages of word embedding and computer vision models. **x**:deep processing, **o**: shallow processing, small black circles: voxels that were negatively explained by both types of models regardless of condition. A few (100 voxels for all subjects, ROIs, and conditions) that have extreme negative variance explained ($<$ -1000) are not shown on the figure.

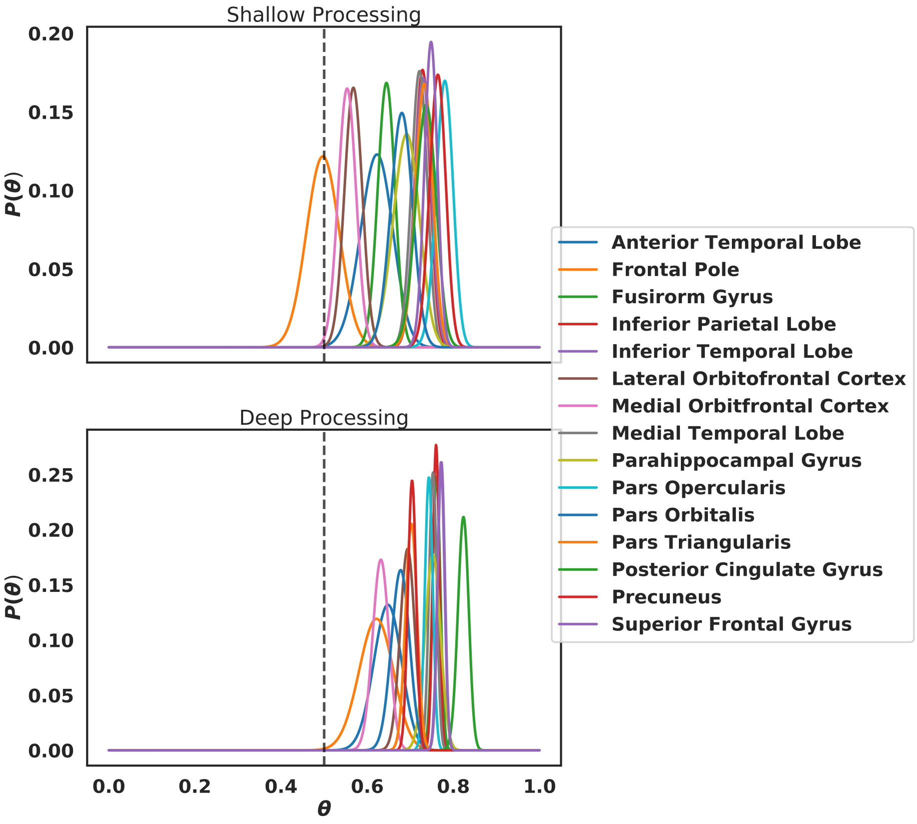

Figure S15: Bayesian inference estimate of how likely a voxel is better explained by a computer vision model than a word embedding model (θ). The prior probability of the above was given by a prior distribution centered at 0.5. For a given ROI and condition, voxels that were better explained by computer vision models were labeled '1' and '0' otherwise. The posterior probability was computed by multiplying the prior probability and the likelihood of '1'. For a given voxel in almost all the ROIs and conditions, it was indeed more likely that computer vision models explained more variance than word embedding models, the only exception being the frontal pole in the shallow processing condition.
